# Impact of enteral nutrition on nitrogen balance in patients of trauma

**DOI:** 10.4103/0974-2700.62101

**Published:** 2010

**Authors:** Sabita Jivnani, Sandhya Iyer, Kabeer Umakumar, M A Gore

**Affiliations:** Department of Surgery, LTMGH & LTMMC, Sion, Mumbai, India

**Keywords:** Enteral nutrition, nitrogen, nutrition assessment

## Abstract

**Background:**

A prospective study of 50 patients of trauma was carried out at a tertiary level trauma center in Mumbai. The aim was to study the hypermetabolic response to trauma and the effect of early enteral feeding and nutritional supplementation in blunting this response in these patients.

**Methods:**

Early enteral feeding was started within 72 h in most patients. The caloric requirement was calculated as per the body weight and a 150: 1 ratio of nonprotein calories to protein was maintained. A 24-h urinary nitrogen loss was estimated and nitrogen balance was calculated on days 1, 3 and 7.

**Results:**

The correlation between the injury severity and the severity of catabolism was also analysed. Urinary nitrogen loss and nitrogen balance were used as parameters to evaluate the hypermetabolic response.

**Conclusions:**

Early (within 72 h) enteral nutritional support blunts this hypermetabolic response to some extent in these trauma patients.

## INTRODUCTION

Trauma is one of the leading causes of death in the age group of 40 years. Profound adverse systemic effects occur as a result of the hypermetabolic response in the first 24–48 h of injury. The hypermetabolic and septic effects of this response can be attenuated by early administration of enteral nutrition. Initiation of enteral nutrition within 72 h,of injury facilitates improved clinical outcomes, decreases the negative nitrogen balance and hence blunts the hypermetabolic response. The goal of nutrition was to minimize nitrogen loss.

## METHODS

This is a prospective study of 50 trauma patients, at a tertiary level trauma center in Mumbai. There were no competing interests in this study.

Patients between age of 18 and 60 years of both sexes admitted in the trauma care unit with a Glasgow Coma Scale (GCS) of 7-13 were included in the study. Patients included those with chronic renal failure and/or serum creatinine >1.5 mg/dl; those on parental nutrition and pregnant females were excluded from the study.

The patients included in the study were divided into two groups:

- Group 1 those having head trauma alone and
- Group 2 those having head trauma with other systemic trauma.

Glasgow Coma Scale and Injury Severity Score (ISS) of these patients were calculated on admission. All patients were subjected to chest X-ray (PA view) and plain CT scan of brain on the day of admission.

The following investigations were carried out for all patients on the day of admission (day 0), and on days 1, 3, 7 and 10 for those who were not lost to follow-up either due to death or discharge. (Random blood glucose level was carried out on the day of admission and repeated only when the patient was a diabetic or when blood glucose levels were >160 mg/dl on admission. Total proteins, serum albumin and serum electrolytes were sent on days 1, 3, 7 and 10).

**Table:** 

**Investigation**	**Expressed in**	**Method**
Hemoglobin	g/dl	Sahlis method
White blood cell count	/mm^3^	Low power microscope
Blood urea nitrogen	mg/dl	Urease method
Serum creatinine	mg/dl	Jaffe's method
Total proteins and serum albumin	g/dl	Biuret method
Serum electrolytes	mEq/dl	Flame photometer
Random blood glucose levels	mg/dl	Glucose oxidase method

A 24-h urine was collected for all patients on days 1, 3 and 7, and 5 ml of urine sample was sent for the following investigations.

**Table:** 

**Investigation**	**Expressed in**	**Method**
24-h urinary urea nitrogen	mg/dl	Urease method
24-h urinary creatinine	mg/dl	Jaffe's method
24-h urinary amino acids	mg/dl	2,4 dinitrobenzene method
24-h urinary uric acid.	mg/dl	Uricase peroxidase method

The daily caloric and protein requirements of these patients were calculated with the help of a nutritionist. The daily caloric requirement was calculated for each patient based on the measurement of 35 kcal/kg/day. Ideal body weight was used for this purpose. Daily protein requirement was calculated as per 1.5 g/kg/day. Approximately 50%,extra protein was added to the daily protein requirement to maintain a ratio of 150 nonprotein kilocalories per 1 g of nitrogen intake. Patients in both the groups were administered the recommended feeds enterally by one of the following four routes:

1. Nasogastric tube feeding
2. Feeding gastrostomy
3. Feeding jejunostomy and
4. Oral feeds

These feeds were started within 72 h of admission; half feeds were introduced on day 3 and full feeds were started by day 4.

Nitrogen balance was calculated as follows:

1. Nitrogen balance (g/day) = Nitrogen intake (g/day) – Total nitrogen output (g/day)
2. Total Nitrogen output (g/day)=24h nitrogen output (g/day)+4 where, 4 is the factor for nitrogen loss through stools, sweat and wounds. It also includes non-urea nitrogen loss.
3. Nitrogen intake (g/day)=Protein intake (g/day)/6.25, where 6.25 is the conversion factor for protein content to nitrogen content.
4. $$\mathrm{Total}\;\mathrm{nitrogen}\;\mathrm{output}\;(g/\mathrm{day})=\frac{24\;\mathrm{hour}\;\mathrm{urinary}\;\mathrm{nitrogen}\;\mathrm{loss}\;\times \;24\;h\;\mathrm{urine}\;\mathrm{output}\;+\;4\;}{100\;000}$$
5. Twenty-four hour urinary nitrogen loss = 24-h urinary urea nitrogen + 24-h urinary creatinine + 24-h urinary amino acids + 24-h urinary uric acid.

The data was recorded in a proforma and was analysed at the completion of study with the help of paired *‘t’* test.

## RESULTS

In our study of 50 patients of trauma, 31 patients (62%) were in the age group of 18–30 years with a male preponderance i.e., M: F ratio of 7.33: 1 [Table 1].

**Table 1:** Age and sex distribution of patients in both the groups

Age group	Group 1		Group 2		Total
			
	Male	Female	Male	Female	
18-30	13	01	15	02	31
31-40	04	00	03	00	07
41-50	02	01	03	00	06
51-60	03	01	01	01	06
Total	22	03	22	03	50

IN BOTH THE GROUPS, MOST OF THE PATIENTS WERE IN THE AGE GROUP OF 18-30 YEARS WITH A MALE : FEMALE RATIO OF 7.33 : 1

Negative nitrogen balance was observed in both the groups on days 1, 3 and 7 [Table 2]. The nitrogen balance in Group 2 patients was more negative than Group 1 patients on days 1, 3 and 7. However, this difference did not reach statistical significance. The nitrogen balance in Group 1 patients improved from-10.95 g/day on day 1 to –6.62 g/day on day 7 (improvement by 4.34 g/day), while that in Group 2 improved from –12.54 g/day on day 1 to –10.11 g/day on day 7 (improvement by 2.42 g/ day). Therefore, the patients of head trauma alone had more improvement in negative nitrogen balance than the patients of head trauma with other systemic trauma even though both groups remained in negative nitrogen balance on day 7 (after starting enteral nutrition). This difference did not reach statistical significance [Table 2].

**Table 2:** Nitrogen loss and change in nitrogen balance on days 1, 3 and 7 in both groups

Day	Group 1		Group 2	
		
	Mean nitrogen loss (g/day)	Change in nitrogen balance (g/day)	Mean nitrogen loss (g/day)	Change in nitrogen balance (g/day)
1	10.95 ± 2.28	–10.95 ± 2.28	12.54 ± 2.35	–12.54 ± 2.35
3	18.13 ± 3.63	–9.71 ± 2.41	21.12 ± 4.49	–13.72 ± 3.73
7	23.88 ± 3.54	–6.62 ± 2.85	27.34 ± 3.46	–10.11 ± 4.36

THE NITROGEN LOSS PROGRESSIVELY INCREASED IN BOTH THE GROUPS, BUT THE NITROGEN BALANCE SHOWED IMPROVEMENT MORE IN GROUP 1 THAN IN GROUP 2 PATIENTS WITH ENTERAL FEEDING

Patients in both the groups had progressively increasing 24-h mean nitrogen losses from day 1 to day 7 despite improvement in their nitrogen balance. This 24-h mean nitrogen loss was greater in Group 2 than Group 1 patients on days 1, 3 and 7. The increase in 24-h mean nitrogen loss on day 7 was more in Group 2 patients (increased by 1480 g/day) as compared to Group 1 patients (increased by 12.93 g/day). However, this difference in increase in the two groups was not statistically significant [Table 2]. (*P*-value calculated by *t* test <0.05.)

The ISSs in Group 1 patients varied from 9 to 16 with a meanISS of 11.8 with a median of 9 and mode of 9. The range of ISS in Group 2 patients was 13-34 with a mean ISS of 19.88 with a median of 20 and mode of 20.24. The mean nitrogen balance on day 1 in Group 1 patients was –10.95 ± 2.28 g/day with a mean ISS of 11.8, while the same in Group 2 patients was –12.54 ± 2.35 g/day with a mean ISS of 19.8 (*P* > 0.05; statistically not significant). This shows that there is more negative nitrogen balance in patients with higher ISSs than in those with lower ISSs [Table 3].

**Table 3:** Mean injury severity score and mean nitrogen balance on day 1 of patients in both the groups

Group	Mean ISS	Range of ISS	Mean nitrogen balance on day 1 (g/day)
1	11.80	9-16	–10.95 ± 2.28
2	19.88	13-34	–12.54 ± 2.35

ISS: INJURY SEVERITY SCORE; MEAN ISS IN GROUP 1 AND GROUP 2 PATIENTS WAS 11.8 AND 19.88, RESPECTIVELY. MEAN NITROGEN BALANCE ON DAY 1 IN GROUP 1 PATIENTS WAS –10.95 ± 2.28 G/DAY AND IN GROUP 2 PATIENTS WAS –12.54 ± 2.35 G/DAY

All the 50 patients in our study received enteral nutrition through various routes. Thirty-three patients from both the groups received nasogastric feeding (15 in Group 1 and 18 in Group 2). Eleven patients from both the groups (5 in Group 1 and 6 in Group 2) were started on oral feeds. Rest of the five patients (four, in Group 1 and one, in Group 2) had gastrostomy feeds, while one patient in Group 1 received jejunostomy feeds. Enteral feeds were initiated within 72 h of admission in all the patients except two,patients in Group 1 and four patients in Group-2. In these patients, enteral feeds could be started only on day 4 or day 5.

The average daily protein and calorie intake in Group 1 patients (52.45 g/day and 1333.68 kcal/day, respectively) were more than the Group 2 patients (46.8 gday and 1185.08 kcal/day respectively) on day 3, but was statistically insignificant. However by day 7, both the groups had almost similar average daily protein and calorie intakes (Group 1 → 107.6 g/day protein intake and 2765.3 kcal/day, Group 2 → 107 g/day protein intake and 2692.3 kcal/day).

The mean nitrogen balance in the patients who could not be started on enteral feeds within 72 h showed a worsening trend on day 3. However, with the introduction of enteral feeds on day 4 or 5, the mean nitrogen balance improved on day 7. This trend was seen in all the patients (2 in Group 1 and 4 in Group 2) in whom enteral feeds could be started only on day 4 or 5 [Table 4].

**Table 4:** Patients in both the groups with delayed nutritional support and their serial nitrogen balance

Group	Case number	Mean nitrogen balance (g/day)		
		
		Day 1	Day 3	Day 7
	2	–8.77	–10.61	–2.78
1	15	–7.60	–18.32	–11.24
	5	–12.50	–28.70	–14.08
	17	–10.71	–16.93	–11.32
2	22	–12.33	–15.71	–7.22
	25	–9.67	*–16.50	–7.81

* THE NITROGEN BALANCE BECAME MORE NEGATIVE ON DAY 3 IN ALL THE SIX PATIENTS IN THE ABSENCE OF ANY NUTRITIONAL SUPPORT BUT SHOWED RECOVERY BY DAY 7 ON STARTING NUTRITIONAL SUPPORT

There were a total of 9 deaths in our study (6 in Group 1 and 3 in Group 2). All the patients who died had GCS range of 7–9. However, no correlation was found between the mean nitrogen balance of patients in both the groups on day 7 with the mortality in our study.

Thirty-two patients (13 in Group 1 and 19 in Group 2) out of 50 underwent surgical intervention in our study (13 patients in Group 1 underwent craniotomies, 16 patients in Group 2 underwent craniotomies and orthopedic interventions and 3 patients in Group 2 underwent emergency laparotomies for blunt abdominal trauma). These patients had a mean nitrogen balance of –8.494 ± 3.78 g/day on day 7. Eighteen patients who did not undergo operative intervention had their mean nitrogen balance –8.139 ± 4.61 g/day on day 7. The difference between mean nitrogen balance of the operated and non-operated groups was not statistically significant [Table 5].

**Table 5:** Mean nitrogen balance on day 7 of operated and non-operated patients

Management	Group	Number of patients	Mean nitrogen balance on day 7 (g/day)
Operative			
Craniotomy	1	13	–6.19
Craniotomy orthopaedic intervention	2	16	–10.461
Laparotomy	2	03	–9.783
Total		32	–8.494 ± 3.78
Non-operative	1	12	–7.08
	2	06	–9.52
Total		18	–8.139 ± 4.61

THE MEAN NITROGEN BALANCE ON DAY 7 IN BOTH THE GROUPS WAS NOT SIGNIFICANTLY ALTERED BY OPERATIVE MANAGEMENT

## DISCUSSION

Trauma ranks as the leading cause of death for individuals up to 40 years of age and third for all age groups. Patients with severe multiple trauma are a challenge in critical care, partly because their primary injuries and subsequent magnitude of organ dysfunction do not appear to follow,predictable patterns readily. We now know that within 24-48 h after trauma, a hypermetabolic response occurs that results in hypercatabolism, hyperglycemia and vascular endothelial instability all of which have profound systemic effects.[1] Research indicates that delaying administration of nutrition to patients of trauma can have potentially lethal effects including septic complications and multiple organ dysfunction syndrome. Although stress hypermetabolism cannot be stopped, its hypercatabolic and septic effects can be attenuated by delivering nutrients enterally. Most nutrition researchers advocate beginning enteral nutrition delivery within 72 h after injury to facilitate improved clinical outcomes.

We conducted a prospective study of 50 patients of trauma admitted at a tertiary level trauma center in Mumbai. Our aim was to study the hypermetabolic response to trauma in these patients and the effectiveness of early enteral nutrition in blunting this hypermetabolic response. Twenty-four hour nitrogen balance is an easily measurable parameter, which is most consistently linked to improved clinical outcome. The overall cost of measuring nitrogen balance is low. Hence, this parameter was chosen in our study to represent the hypermetabolic response.

In our study, most patients (31-62%) were in the age group of 18-30 years with a male preponderance (7.33:1). These figures correlated well with National Trauma Data Bank figures. It maintains records of trauma centers all over the United States where the peak distribution of trauma patients was recorded in the 17-24 year age group. This data bank shows a male-female ratio of 3:1.

In our study, all the patients in both the groups were in negative nitrogen balance from day 1 (–10 to 95 g/day in Group 1 and –12 to 54 g/day in Group 2) and continued to remain in negative nitrogen balance even on day 7 after injury (–6 to 61 g/day in Group 1 and –10 to 11 g/day in Group 2). This shows that patients of trauma undergo a hypermetabolic phase immediately following trauma and continue to remain hypermetabolic even at the end of one week despite adequate enteral nutritional support. Nygren *et al.* have found similar nitrogen balance levels approximately –10 g/day on the first day in trauma patients.[2]

In Group 1 patients, the mean nitrogen balance improved from –10.95 g/day on day 1 to –6.61 g/day on day 7. This improvement in mean nitrogen balance in Group 1 patients was by 4.34 g/day. In Group 2 patientstoo, the mean nitrogen balance improved from –12.54 g/day on day 1 to –10.11 g/day on day 7, i.e., improvement by 2.42 g/day. This indicates that in our study, patients with head trauma alone had better improvement in mean nitrogen balance as compared to patients of head trauma with other systemic trauma after starting early enteral nutritional support. Moore has also shown that there is an improvement in nitrogen balance of trauma patients with early enteral feeding in both head trauma as well as polytrauma patients.[3] However, this difference did not reach statistical significance in our study.

Another interesting fact was that the mean 24-h nitrogen loss continued to increase in all the patients in both the groups (i.e, from 10.95 g/day on day 1 to 23 g/day on day 7 in Group 1 patients and from 12.54 g/day on day 1 to 27.3 g/day on day 7 in Group 2 patients). Again here, the patients of head trauma had comparatively less nitrogen loss than their counterparts even though the difference was not significant (increase in mean nitrogen losses from day 1 to day 7 in Group 1 patients was 12.93 g/day and in Group 2 patients, it was 14.80 g/day). Despite the worsening mean nitrogen loss, both groups showed an improvement in their negative nitrogen balance. This underscores the importance of early enteral nutritional support in these patients.

The mean ISS in Group 1 patients was 11.8 with a range from 9 to 16. As expected, the mean ISS in Group 2 patients was higher i.e., 19.88 with a range from 13 to 34. The mean nitrogen balance on day 1 in Group 1 patients with meanISS of 11.8 was –10.95 ± 2.28 g/day, while the same on day 1 in Group 2 patients was –12.54 ± 2.35 g/day with a mean ISS of 19.88. This difference even though not statistically significant shows that ISS has a definite correlation with the nitrogen balance in trauma patients. Nataloni *et al.* have shown that ISSshows a negative linear correlation with the nitrogen balance in trauma patients that is, a higher injury severity results in a more negative nitrogen balance.[4] Jeevanandam *et al.* have found severe nitrogen losses and a prolonged period of negative nitrogen balance in patients with higher ISS.[5]

Most of the patients in both the groups were started on enteral feeding within 72h of admission through one of the following routes, viz nasogastric tube feeds,,oral feeds, gastrostomy or jejunostomy feeds. Daily caloric requirement was calculated as per 35 kcal/kg/day and daily protein requirement was calculated as per 1.5g/kg/day. Fifty percent extra protein was added to the normal daily requirement and a ratio of 150 non-protein kilocalories per 1 gram of nitrogen intake was maintained. Ideal body weight was used for calculating daily caloric and protein requirements. The average daily caloric and protein intake in both the groups were comparable on day 7 (2765.30 kcal/day in Group 1 patients and 2692.36 kcal/day in Group 2 patients, while 107.6 g protein/day in Group 1 patients and 107 g protein/ day in Group 2 patients).

In two and four patients in Group 1 and 2, respectively, enteral nutrition could be initiated only on day 4 or 5. The 24-h nitrogen balance in all the six patients worsened on day 3 in the absence of any nutritional support. However, with the initiation of enteral feeding on day 4 or 5, the nitrogen balance in all the six patients showed improvement. This highlights the importance of starting early enteral nutritional support in trauma patients. The value of early enteral nutritional support has been proved by many large randomized controlled trails and a metaanalysis was done by David *et al.* and the results were formulated as guidelines for nutritional support.[6] The advantage of early enteral feeding in blunting the catabolic response by improving the nitrogen balance of trauma patients has been studied by various researchers, such as Frankenfield in 1997,[7] Moore *et al*. in 1991,[8] Young in 1987,[9] Heyland in 1999[10] and recently by Falcao *et al*. in 2000[11] and many more trials are going on.

Metaanalysis of randomized controlled trails done by Heyland[10] in 1999 and more recently by the EAST group[6] also recommend early nutritional support whenever possible by enteral route in all trauma patients.

In Group 1 patients there were 6 deaths out of 25 patients; a mortality of 24%. In Group 2 patients, there were 3 deaths out of 25 patients; a mortality of 12%. An attempt was made to correlate the worsening or improvement of nitrogen balance of the survivors and the non-survivors. However, in our study, the improvement or worsening of nitrogen balance failed to show any correlation with the final outcome. Similar results were seen by Heyland.[10]

On further analysis, it was found that in both the groups, all the nine patients who died had GCS ranging from 7 to 9 on admission, while none of the patients with a GCS ranging from 10 to 13 had mortality. The poor outcome in these patients with lower GCS can be attributed to the primary severe cerebral injury. Feldman *et al*. in 1993[12] and Wilson in 2001[13] have also showed a correlation between GCSof less than 8 with poor outcome.

In our study, 32 patients underwent surgical intervention for their head trauma or other systemic trauma. Thirteen patients in Group 1 underwent craniotomies for their head trauma. Sixteen patients in Group 2 underwent craniotomies with or without orthopedic interventions like amputation, external fixation or open reduction and internal fixation etc. for their polytrauma while 3 patients in Group 2 underwent emergency laparotomies for blunt abdominal trauma.

The mean nitrogen balance of operated patients on day 7 was –8.49 g/day ± 3.78, whereas that of conserved patients was –8.139 g/day ± 4.61. This difference was not statistically significant. This indicates that surgical intervention did not adversely affect the nitrogen balance of the patients in our study. Nast-Kolb *et al*.[14] have also not found any significant difference in the nitrogen balance of operated and non-operated trauma patients.

Thus, the major impact of nutritional support through the enteral route has now been recognized in aiding host defense and repair mechanisms in addition to preventing malnutrition and its complications. However, much research is required in this field with larger number of patients and to be carried out on a prolonged basis to understand the interrelationships amongst the variables in heterogenic and complex population affected by trauma.

### Strengths of the study

The aim of this study was to evaluate the hypermetabolic response to trauma and the effectiveness of early nutritional support in blunting this response. S 24-h urinary nitrogen estimation is an easily measurable, cost effective investigation that has been consistently linked to clinical outcome and hence this was chosen as the parameter of hypermetabolic response. Patients in this study showed increasing nitrogen losses but showed an improvement in the negative nitrogen balance. The extent of nitrogen losses correlated with the ISS. These results were comparable to other studies.

Even though the results of early enteral nutrition has been proved and published many times, this article is an attempt to evaluate the response to early enteral feeds in patients of trauma in a general hospital in the Indian scenario. Indian patients differ from Western counterparts in types of insult, metabolic response to trauma, pre-trauma nutritional status and underlying systemic diseases. We formulated our own guidelines and protocols to evaluate this group of patients.

### Limitations of the study

The European society for clinical nutrition and metabolism guidelines mention that early enteral nutrition should be stared in the first 24 h. Many patients in our study were being investigated and evaluated for operable lesions. Hence, enteral nutrition was started after surgical treatment of these or after a final decision regarding nonoperative treatment was taken. Even when there was a delay in initiating enteral nutrition, there was a reduction in the negative nitrogen balance. Due to the small study sample, many of the changes were not statistically significant. Similarly due to the small sample, a focused and consistent population could not be chosen. Stability of caloric intake could not be achieved due to erratic tolerance to tube feeds by some patients. Serum transferrin and prealbumin estimations were not done as these were not performed by the hospital laboratory.

### Future directions

More studies with larger sample of patients need to be undertaken with longer periods of monitoring to study the complex metabolic changes in injured patients, nutritional needs and to determine prognostic factors that will aid in recognizing those at highest risk of wasting and malnutrition.

## CONCLUSIONS

From our prospective study of 50 patients of trauma we conclude that

1. Trauma induces a hypermetabolic response, which leads to a state of persistent negative nitrogen balance.
2. Early (within 72 h) enteral nutritional support blunts this hypermetabolic response to some extent in these trauma patients.
3. The greater the severity of injury the more severe is the catabolic response to injury.
4. Glasgow Coma Scale is a predictor of poor outcome in trauma patients irrespective of patients’ nutritional status.
5. Surgical intervention in these hypermetabolic patients does not affect nitrogen balance significantly.

Thus, it is important to initiate early enteral nutrition in the trauma patients to blunt their hypermetabolic response and improve their nitrogen balance. More such studies with greater number of patients are needed to establish the impact of nitrogen balance on outcome of these patients of trauma.
